# Three-dimensional kinematic analysis of ankle, knee, hip, and pelvic rotation during gait in patients after anterior cruciate ligament reconstruction - early results

**DOI:** 10.1186/s12891-015-0726-8

**Published:** 2015-09-28

**Authors:** Andrzej Czamara, Iga Markowska, Magdalena Hagner-Derengowska

**Affiliations:** The College of Physiotherapy in Wroclaw, ul. Kościuszki 4, 50-038 Wroclaw, Poland; The Center of Rehabilitation and Medical Education, ul. Kościuszki 4, 50-038 Wroclaw, Poland; The Chair and Department of Clinical Neuropsychology, Collegium Medicum, The Nicolaus Copernicus University in Torun, ul. Jagiellonska 15, 85-067 Bydgoszcz, Poland; The University in Bydgoszcz, ul. Jana Karola Chodkiewicza 30, 85-064 Bydgoszcz, Poland

**Keywords:** Joint rotation, Anterior cruciate ligament reconstruction (ACLR), Postoperative management

## Abstract

**Background:**

The goal of this study was to biomechanically assess tibial rotation in the knee joint simultaneous changes in rotation of large joints of the lower limbs and pelvis during gait in patients during early postoperative stages following anterior cruciate ligament (ACLR) reconstruction.

We hypothesized that tibial rotation is associated with changes in rotation of the large joints of the lower limbs and the pelvis during gait in patients after ACLR reconstruction.

**Methods:**

The patients were divided into two groups. The ACLR group (*n* = 32 males) underwent primary ACLR in one leg and postoperative physiotherapy. The control group (*n* = 30 males) had no knee injuries. After clinical assessment in both groups, the values of kinematic parameters of foot, tibial, femoral, and pelvic rotation were measured during gait on a flat surface using the three-dimensional BTS Smart System. In the ACLR group, measurements were taken during the 4th, 9th, and 14th weeks of postoperative physiotherapy. The results of the ACLR group were compared with those of the control group.

**Results:**

During gait, between the 9th and 14th weeks following ACLR, there are normal values of foot, tibia, and pelvic rotation in the operated legs compared with results obtained from un-operated legs and the control group.

**Discussion:**

Analysis of rotations occurring only in knee joints does not reflect all of the multiarticular disorders of gait kinematics. The study also suggests that analyzing tibial rotation in the knee joint with simultaneous changes in rotation in large joints of the lower limbs provides better opportunities than singular analysis of rotation in the knee joint for the assessment of disorders in gait kinematics.

**Conclusions:**

In gait, at the maximal extension of the knee during preparation for the stance phase, external hip rotation patterns have not been fully restored 14 weeks after ACLR.

## Background

Biomechanical assessment of gait kinematic parameters has been carried out in patients before and after anterior cruciate ligament reconstruction (ACLR) [1, 2]. Analysis of gait kinematics is included in assessment of the effects of accelerated rehabilitation after ACLR [3]. The behaviour of kinetic, kinematic, and electromyographic parameters during gait has been compared in subjects with chronic ACL lesions and those after ACLR [4, 5, 6]. Additionally, early analysis of gait kinematic parameters and asymmetry indices has been carried out in ACLR patients [7, 8]. The gait kinematic test is part of complex functional assessment of the knee joint in patients after ACLR. This test involves assessment of the developed muscle strength and physical fitness of the entire motor organ [9]. Better rotational stability of the tibia has been shown in ACLR patients during gait compared with that in patients with chronic ACL deficiency who had repeated episodes of joint rotational instability, which can contribute to the development of degenerative disease of the knee joint [10]. Nevertheless, the kinematic parameters of gait are not fully restored in ACLR patients compared with control values [11]. To date, there are no reports on simultaneous changes in gait parameters between 4th and 14th weeks after ACLR, in the transverse plane of the knee, associated with changes in the neighbouring joints of the lower limbs and the pelvis.

Therefore, the goal of this study was to perform biomechanical assessment and analysis of tibial rotation in the knee joint with simultaneous changes of rotation in the large joints of the lower limbs and the pelvis in patients shortly after ACLR.

## Methods

Sixty two men were divided into two groups. Thirty-two males underwent primary anterior cruciate ligament reconstruction of the knee - the ACLR group (20 right legs, 12 left legs) in one leg. The control group included 30 males with no injuries and no diseases of the knee joints and no diseases of the lower extremities. The ACLR group underwent double-bundle reconstruction (DB ACLR) using the position method (*n* = 13) and single-bundle reconstruction (SB ACLR) using the Endobutton method (*n* = 19). In 10 patients, a concomitant lesion of the medial meniscus was noted (shaving was performed), and/or first and second degree chondromalacia of the femoral condyle according to the International Cartilage Repair Society. These conditions did not require surgery. The study was conducted according to the ethical guidelines and principles of the Declaration of Helsinki. The study protocol was approved by the ethics committee of the College of Physiotherapy in number 1/2012.

### Orthopaedic examinations

Initially, 48 patients after ACLR participated in the study. All patients underwent orthopaedic examination. Orthopaedic examination was also carried out on 30 healthy volunteers with a view to their inclusion in the control group. An orthopaedist carried out anamnesis. This involved collecting information on the patient’s subjective assessment of stability in the operated joint and pain based on the visual-analogue scale. The clinical tests for the operated knee and the un-operated knee included Lachman’s test, the anterior drawer test, stability of the posterior cruciate ligament, collateral ligaments, and menisci, and patellofemoral joint tests. The Q-angle, the axis of the lower limb, and range of movement and circumference of the knee were evaluated [12].

The inclusion criteria in both groups were: regular physical activity three to four times a week (score 7 according to the Tegner Activity Scale; in the ACLR group before ACL damage) and clinically stable knees (anterior translation of less than 3 mm). Additionally, inclusion criteria in the ACLR group were as follows: 1) primary ACLR in one leg with or without medial meniscus shaving and/or with cartilage lesions that did not require surgery; 2) the time between total tearing of the ACL and surgery was no longer than 4 months; 3) the patient consented to participate in the study and to undergo treatment; 4) use of anticoagulative, anti-inflammatory, and analgesic medications for no longer than 4 weeks after ACLR; 5) regular physiotherapy was carried out an average of four times a week during a minimum of 14 weeks with the same physiotherapist in the rehabilitation centre where the study was conducted; 6) participation in all of the three analysis of gait; 7) no pain during tests and during 2 consecutive days following the tests; 8) no postoperative complications; 9) no differences between legs in the range of movement and circumference of the knee joint during the 14th week postoperatively. Inclusion criteria for the control group: 1) voluntary participation in the study; 2) no differences between limbs in the range of movement and knee joint circumference.

The exclusion criteria in both groups were the following clinical complications: 1) additional injuries to the knee joint, articular cartilage, and ligaments requiring surgery; 2) injuries of the neighbouring joints of the lower limbs and/or the pelvic and bone fractures; 3) deformations; 4) malalignment of the axis and length of the lower limbs and other orthopaedic surgeries. We excluded five patients with concomitant hip joint injuries, four with postoperative complications (persistent swelling or pain), three who underwent only two gait analyses, and four who withdrew from physiotherapy between the 6th and the 8th weeks following surgery.

No significant between ACLR group and control group differences in age, body mass, and body height were found (Table 1).

**Table 1:** Characteristics of male patients in the ACLR and control groups

Group	Age (years)	Body height (cm)	Body mass (kg)	Dominant legs
ACLR group (*n* = 32)	28.65 ± 8.57	181.25 ± 8.77	81.90 ± 11.17	28 right ± 4 left
Control group (*n* = 30)	25.93 ± 4.57	180.86 ± 7.20	80.46 ± 12.30	28 right ± 2 left
p	0.631	0.305	0.348	

*p* level of significance

### Postoperative physiotherapy

The ACLR group was subject to a minimum of 14 weeks of physiotherapy, for an average of four times a week at the rehabilitation centre [13]. Physiotherapy was carried out by an experienced physiotherapist, based on referral from an orthopaedic surgeon. Additionally, patients were instructed and advised to exercise at home [13].

### Recording and measurement of kinematic data

Recording and measurement of kinematic parameters was performed during gait for individual lower limb joints and the pelvis. The BTS Smart (BTS Bioengineering, Milan, Italy) optoelectric system for three-dimensional (3D) recording and analysis of movements was used. Six infrared light camcorders (sampling frequency of 120 Hz) and two piezoelectric Kistler platforms (sampling frequency of 960 Hz) were used. The devices were calibrated prior to the study [14]. Anthropometric measurements of the right side of the body followed by measurements of the left side of the body were taken. Body height (cm), body mass (kg), the width and depth of the pelvis, the width of the knee joints and ankle joints, and the length of the lower limbs were measured (cm). After skin cleansing, 23 markers were placed on the patient’s skin on both legs, at the level of the tuber calcanei, the fifth metatarsal head, the centre of the ankle joint laterally, half the length of the fibula, the fibular head and the lateral condyle of the femur, half the length of the femur, the area of the femoral trochanter, the antero-superior iliac spine, and at the level of the S1 vertebra [15]. Measurements of the kinematic parameters during gait were carried out three times: at the end of the 4th (first measurement), 9th (second measurement) and 14th (third measurement) weeks after ACLR postoperatively. In the control group, the test was carried out twice. None of the patients reported pain on the day of the study. Each test began with a 10-second static measurement on the platform. During the measurement, each patient stood comfortably with their upper limbs along the trunk. The patient then walked six times along the measurement path at a moderate speed (natural speed; walking speed was about 1 m/s) for a distance of 7–8 m, without shoes, starting each time with the left leg. Each walk was performed in identical conditions. The data collected during the test were processed using BTS software where a model was developed from single points, analogical to the original Davis pattern [14, 15]. Among all of the recorded walks, the two best walks were selected, as determined by stepping on the piezoelectric platform and all of the markers on the patient’s body were read during the gait. Six episodes were recorded during the gait as follows: (1) angle value, which was the angle during the first heel contact with the floor (KHS); (2) maximal flexion of the knee of in the stance phase (K1); (3) maximal extension of the knee during a single stance (K2); (4) when the toes were lifted off the floor (KTO); (5) maximal flexion of the knee joint during the swing phase (K3); (6) and maximal extension during preparation for the stance phase (K4). Moreover, the values of kinematic and kinetic parameters were recorded and measured in the transverse planes. Movements of internal rotation (IR) and external rotation (ER) of the ankle joints, in the knee joints, in the hip joints, and in the pelvis were measured and expressed in degrees (°). ER values are shown with a minus (−) sign. Biomechanical measurements were performed by one person.

### Test-retest reliability

Test-retest reliability was performed in the control group to assess the reliability of the obtained values of kinematic rotation in the transverse plane in the joints of the ankle, knee, hip, and pelvis during gait using the BTS system. Between the first and second measurements were 2 days break, and 3D registration of kinematic rotation parameters of the lower limb joints and pelvis was carried out, in accordance with the above-mentioned registration procedure. The intraclass correlation coefficient (Shrout and Fleiss model 2) were calculated to compare the data between sessions in the test–retest assessment [16]. The following guidelines, described by Cicchetti and Sparrow, were used to assess reliability coefficients: less than 0.40 was considered poor, 0.40–0.59 was considered fair, 0.60–0.74 was considered good, and 0.75 or greater was considered excellent [17].

In the present study ICC test results for the pelvis rotation between the first and second tests ranged from 0.713 to 0.909 (most results ranged from 0.800 to 0.909). For the hip joint, the ICC result in most phases of the gait was above 0.900. In one case, the ICC was 0.534 for the left extremity during gait in the first contact with the ground heel (KHS phase). For the knee joint, the ICC was over 0.950. The ICC values for the ankle were in the range of 0.770–0.870. For mechanical accuracy mean difference between two tests was calculated for the pelvis (0.08^0^ – 0.41^0^), hip (0.06^0^ – 0.68^0^), knee (0.14^0^ – 0.49^0^), ankle (0.49^0^ – 0.57^0^).

### Statistical analysis

The mean value and standard deviation were measured in all groups. The Shapiro–Wilk test [18, 19] was carried out to study the distribution. In order to compared the operated limbs to un-operated limbs in the ACLR group, the parametric *t*-test with 95 % CI or the non-parametric Wilcoxon test were applied. The parametric t-tests were performed for the operated limb in the first measurement phase K1,K2,KTO,K3; in the second and third measurement carried out for all phases (KHS,K1,K2,KTO,K3,K4). Non-parametric tests were performed for the operated limb in the first measurement phase KHS and K4. For healthy limbs parametric tests were performed: in the first measurement for all phases (KHS,K1,K2,KTO,K3,K4); in the second measurement for phase K2; in the third measurement for phase KTO and K3. Non-parametric tests for healthy limbs were performed: in the second measurement for KHS,K1,KTO,K3,K4; in the third measurement for phase KHS,K1,K2,K4. One-way ANOVA was used to assess the significance of differences between the operated limb of the ACLR group, the right and the left legs in the control group. The ANOVA revealed no statistically significant differences, thus the post-hoc tests were not needed.

## Results

Small and mostly statistically insignificant external rotation (ER) disorders during gait were observed in the ankle joint of the operated legs (Table 2). During the first measurement, in the operated knee, a significantly excessive external tibial rotation (ETR) was observed compared with the un-operated knee from the K1 to K3 phases (Table 2). In the second and third tests, we found that the excessive ETR at the operated side was reduced, but this was not significantly different from the un-operated side. The third test showed smaller values of ETR at the operated side compared with those obtained from un-operated knees. The differences in ETR ranged from 0.4 to 1.5°. In the operated knees, there was a significant difference in ER between one and two measurements for phases K1, K2, and KTO. In phase K3, there was also a significant difference between measurements one and two and between one and three. There was no significant difference between measurements for un-operated limbs (Table 2).

**Table 2:** Values of ankle and knee rotation for each phase of gait cycle in the ACLR group

	Rotation for subsequent phases of the gait cycle		First measurement	Second measurement	Third measurement	ANOVA
Ankle	KHS	Operated leg	−11.4 ± 6.61	−7.81 ± 11.82	−9.69 ± 8.38	0.285
		Un-operated leg	−10.53 ± 8.71	−11.54 ± 6.03	−10.12 ± 7.55	0.741
	P		0.948	0.076	0.799	
	K1	Operated leg	−13.18 ± 7.30	−9.28 ± 11.85	−10.41 ± 8.53	0.241
		Un-operated leg	−10.90 ± 8.80	−10.60 ± 6.56	−9.53 ± 7.36	0.752
	P		0.121	0.395	0.091	
	K2	Operated leg	−12.57 ± 9.17	−11.40 ± 11.55	−12.99 ± 8.18	0.796
		Un-operated leg	−12.79 ± 8.73	−13.39 ± 6.50	−12.98 ± 7.61	0.950
	P		0.717	0.881	0.525	
	KTO	Operated leg	−8.61 ± 10.15	−5.03 ± 8.37	−5.03 ± 8.43	0.192
		Un-operated leg	−11.15 ± 11.04	−6.25 ± 4.20	−7.62 ± 9.26	0.071
	P		0.667	0.875	0.175	
	K3	Operated leg	−13.80 ± 8.94	−11.49 ± 10.92	−11.51 ± 10.93	0.591
		Un-operated leg	−16.00 ± 10.98	−11.69 ± 6.62	−12.45 ± 10.19	0.156
	P		0.667	0.472	0.786	
	K4	Operated leg	−12.00 ± 6.44	−9.35 ± 12.60	−10.38 ± 9.37	0.553
		Un-operated leg	−10.39 ± 8.92	−13.06 ± 6.75	−11.58 ± 7.67	0.397
	P		0.501	0.026	0.472	
Knee	KHS	Operated leg	−20.10 ± 8.67	−15.58 ± 8.22	−17.79 ± 11.14	0.164
		Un-operated leg	−17.16 ± 10.69	−16.47 ± 8.32	−19.32 ± 8.01	0.427
	P		0.282	0.830	0.494	
	K1	Operated leg	−17.21 ± 8.07*	−11.40 ± 8.14*	−12.45 ± 12.18	0.041 ^(1–2)^
		Un-operated leg	−10.40 ± 11.97	−8.46 ± 8.90	−11.54 ± 8.80	0.464
	P		0.017	0.066	0.683	
	K2	Operated leg	−16.92 ± 8.92*	−10.92 ± 7.84*	−13.48 ± 11.52	0.046 ^(1–2)^
		Un-operated leg	−11.27 ± 11.82	−11.59 ± 8.80	−14.76 ± 9.56	0.319
	P		0.034	0.694	0.568	
	KTO	Operated leg	−15.80 ± 8.32*	−9.27 ± 9.19*	−9.99 ± 11.84	0.018 ^(1–2)^
		Un-operated leg	−10.09 ± 10.66	−8.39 ± 8.26	−11.30 ± 7.37	0.422
	P		0.031	0.580	0.528	
	K3	Operated leg	−17.05 ± 9.33*	−8.65 ± 10.55*	−9.30 ± 13.30*	0.005 ^(1–2;1–3)^
		Un-operated leg	−10.92 ± 11.47	−7.43 ± 10.38	−10.08 ± 8.77	0.368
	P		0.019	0.483	0.724	
	K4	Operated leg	−21.18 ± 8.63	−14.88 ± 9.53	−17.91 ± 12.36	0.055
		Un-operated leg	−16.80 ± 10.65	−16.00 ± 8.58	−19.34 ± 8.45	0.329
	P		0.110	0.515	0.532	

^*^- level of significance (*p* <0.05); (−) – external rotation; movements of internal rotation (IR) and external rotation (ER) were measured and expressed in degrees (^0^)

The first measurement showed significantly higher values of IR of the femur in the operated leg for all phases (KHS to K4) of the gait cycle compared with the values obtained from the un-operated legs (*p* <0.001). In the un-opearated joints, a compensatively larger ER of the thigh compared with operated the hip joints was observed (Table 3). In the second measurement, despite an improvement, IR of the thigh in the operated leg was maintained at a significantly higher level for all of the gait phases (from *p* <0.019 to *p* <0.001) compared with the un-operated side where excessive ER was reduced. In the third measurement, further reduction in IR values was observed for the gait phases from KHS to K3, which resulted in a lack of significant difference (Table 3). However, during maximal extension of the operated leg in the K4 phase (preparation for stance phase), ER of the thigh was still 4° smaller compared with that of the un-operated side (*p* = 0.043). ANOVA also showed that most of the changes were significant for hip rotation on the side of the operated knee for all phases of the gait cycle. This was evident for the phases KHS, KTO, and K4 when the first measurement was compared with the second and third measurements (Table 3). In the first measurement, analysis of pelvic kinematics at the side of the operated leg showed a significant reduction in IR at the KHS and K1 phases and excessive ER at the phases K2, KTO, K3, and K4 compared with the un-operated side (from *p* <0.011 to *p* <0.001). The second and third measurements showed similar values of pelvic rotation at the operated side to those obtained from the un-operated side, with no significant difference (Table 3).

**Table 3:** Values of hip and pelvic rotation for each phase of the gait cycle in the ACLR group

	Rotation for subsequent phases of the gait cycle		First measurement	Second measurement	Third measurement	ANOVA
Hip	KHS	Operated leg	7.75 ± 9.64*	1.43 ± 10.14*	−0.78 ± 10.32*	0.003 ^(1.2;1–3)^
		Un-operated leg	−5.96 ± 8.97	−5.60 ± 8.07	−4.12 ± 8.35	0.656
	P		0.001	0.001	0.122	
	K1	Operated leg	9.19 ± 7.81*	5.21 ± 9.13	3.45 ± 9.05*	0.029 ^(1–3)^
		Un-operated leg	−4.90 ± 9.82*	−2.90 ± 7.03	0.30 ± 7.73*	0.045 ^(1–3)^
	P		0.001	0.001	0.132	
	K2	Operated leg	10.28 ± 8.13*	6.86 ± 7.97	4.45 ± 7.65*	0.016 ^(1–3)^
		Un-operated leg	−3.55 ± 9.86	0.09 ± 8.26	2.71 ± 8.29	0.020 ^(1–3)^
	P		0.001	0.001	0.313	
	KTO	Operated leg	10.56 ± 9.56*	5.03 ± 8.05*	4.20 ± 7.90*	0.007 ^(1–2;1–3)^
		Un-operated leg	−1.24 ± 12.58	0.85 ± 7.59	3.67 ± 8.95	0.144
	P		0.001	0.019	0.770	
	K3	Operated leg	12.72 ± 9.32*	7.75 ± 9.57	5.35 ± 9.40*	0.008 ^(1–3)^
		Un-operated leg	−1.33 ± 12.54	0.74 ± 8.00	2.96 ± 9.12	0.238
	P		0.001	0.001	0.177	
	K4	Operated leg	6.29 ± 10.11*	−1.01 ± 10.64*	−2.72 ± 11.02*	0.002 ^(1–2;1–3)^
		Un-operated leg	−7.79 ± 9.26	−8.72 ± 8.40	−6.95 ± 8.08	0.713
	P		0.001	0.001	0.043	
Pelvis	KHS	Operated leg	0.59 ± 4.63*	2.57 ± 3.91	3.95 ± 3.45*	0.005 ^(1–3)^
		Un-operated leg	4.82 ± 5.23	4.59 ± 3.58	4.38 ± 3.89	0.919
	P		0.011	0.081	0.668	
	K1	Operated leg	1.05 ± 4.48	2.50 ± 3.44	3.01 ± 2.94	0.093
		Un-operated leg	5.77 ± 5.42	3.81 ± 3.47	3.72 ± 3.87	0.108
	P		0.006	0.232	0.475	
	K2	Operated leg	−1.63 ± 4.78	−0.99 ± 3.74	−1.09 ± 3.53	0.791
		Un-operated leg	3.01 ± 4.87	0.34 ± 3.08	−0.94 ± 3.85	0.001 ^(1–2;1–3)^
	P		0.003	0.196	0.963	
	KTO	Operated leg	−6.54 ± 4.64*	−3.95 ± 3.47*	−4.31 ± 3.78	0.022 ^(1–2)^
		Un-operated leg	−1.22 ± 3.99	−2.59 ± 2.80	−3.18 ± 3.33	0.067
	P		0.001	0.145	0.285	
	K3	Operated leg	−5.84 ± 4.47	−4.20 ± 3.42	−3.84 ± 3.44	0.088
		Un-operated leg	−1.19 ± 4.26	−2.98 ± 2.69	−3.63 ± 2.91	0.014 ^(1–3)^
	P		0.003	0.199	0.841	
	K4	Operated leg	−0.65 ± 5.42*	2.06 ± 3.20*	3.78 ± 4.36*	0.001 ^(1–2;1–3)^
		Un-operated leg	4.61 ± 5.12	3.43 ± 3.61	3.78 ± 3.72	0.519
	P		0.003	0.161	0.786	

^*^- level of significance (*p* <0.05); (−) – external rotation; movements of internal rotation (IR) and external rotation (ER) were measured and expressed in degrees (^0^)

ANOVA analysis of the ACLR group showed that the third measurement of foot, tibia, hip, and pelvic rotation values obtained from the operated legs were similar to those obtained for the left and right joints of the lower limbs in the control group (not significant, Table 4).

**Table 4:** Comparison of results between operated leg from the third measurement and left and right in the control group

Rotation for subsequent phases of the gait cycle		KHS	K1	K2	KTO	K3	K4
Pelvis	ACLR group Operated leg	3.95 ± 3.45	3.01 ± 2.94	−1.09 ± 3.53	−4.31 ± 3.78	−3.84 ± 3.44	3.78 ± 4.36
	Control group Right leg	3.31 ± 3.31	2.27 ± 2.67	−0.38 ± 2.78	−3.08 ± 2.68	−3.23 ± 2.70	3.77 ± 3.19
	Control group Left leg	3.49 ± 2.77	3.20 ± 2.93	−0.21 ± 3.13	−3.62 ± 2.64	−3.63 ± 3.02	2.97 ± 3.15
	ANOVA p	0.709	0.469	0.239	0.085	0.349	0.571
Hip	ACLR group Operated leg	−0.78 ± 10.32	3.45 ± 9.05	4.45 ± 7.65	4.20 ± 7.90	5.35 ± 9.40	−2.72 ± 11.02
	Control group Right leg	−0.26 ± 9.48	3.40 ± 8.84	4.99 ± 9.60	4.29 ± 9.82	1.47 ± 10.64	−3.77 ± 10.39
	Control group Left leg	−1.92 ± 12.43	1.60 ± 12.03	3.26 ± 12.20	2.17 ± 12.43	−0.37 ± 13.89	−5.23 ± 14.24
	ANOVA p	0.394	0.631	0.316	0.239	0.676	0.457
Knee	ACLR group Operated leg	−17.79 ± 11.14	−12.45 ± 12.18	−13.48 ± 11.52	−9.99 ± 11.84	−9.30 ± 13.30	−17.91 ± 12.36
	Control group Right leg	−21.14 ± 9.97	−14.31 ± 8.59	−16.78 ± 8.45	−13.69 ± 8.94	−11.26 ± 9.42	−21.18 ± 10.28
	Control group Left leg	−18.91 ± 7.69	−12.15 ± 6.52	−14.04 ± 6.02	−10.43 ± 5.71	−9.09 ± 7.80	−18.91 ± 8.13
	ANOVA p	0.831	0.718	0.787	0.658	0.136	0.712
Ankle	ACLR group Operated leg	−9.69 ± 8.38	−10.41 ± 8.53	−12.99 ± 8.18	−5.03 ± 8.43	−11.51 ± 10.93	−10.38 ± 9.37
	Control group Right leg	−11.07 ± 8.11	−12.75 ± 6.96	−15.79 ± 6.62	−10.02 ± 9.12	−15.06 ± 9.40	−12.23 ± 10.07
	Control group Left leg	−11.09 ± 6.30	−11.72 ± 6.63	−15.16 ± 7.12	−7.99 ± 8.85	−11.95 ± 10.57	−12.62 ± 6.92
	ANOVA p	0.412	0.473	0.918	0.435	0.927	0.403

^*^- level of significance (*p* <0.05); (−) – external rotation; movements of internal rotation (IR) and external rotation (ER) were measured and expressed in degrees (^0^)

## Discussion

To date, there has been no comparative analysis of tibial rotation kinematics during gait with simultaneous changes in the rotation of the feet, tibia, femur, and pelvis in patients between the 4th and 14th week after ACLR. The research presented in our study provides new and important information on rotational gait disorders in large joints of the lower limbs and pelvis in patients examined during the 4th, 9th and 14th weeks of postoperative physiotherapy after ACLR.

During the 4th week after ACLR in the operated knee, we found a significantly excessive external tibial rotation (ETR) compared with the un-operated knee from the K1 to K3 phases. In the 9th, and in the 14th weeks after ACLR, the excessive ETR at the operated side was reduced and finally in the 14th week the values of ETR at the operated side were similar to un-operated knees. Claes *et al.* [20] found that 6 months after ACLR tibial rotational was fully restored during plain walking. Gao and Zheng [11] showed that the kinematic parameters of gait are not fully restored in patients after ACLR compared with control values. Czamara *et al.* [21] noted that in the SB ACLR group, more disorders of rotation kinematics were noted in large joints of the lower limbs during gait in comparison with the DB ACLR and control groups. It was also found that 14 weeks of postoperative physiotherapy were not enough to fully restore rotation kinematics in joints of the lower limbs during gait in both groups [21]. However, very few group members were analysed in the quoted research.

It should be noted that the results of the present study show that in the ACLR group, most of the disorders, as well as the most persistent disorders, were found during gait in femoral rotation of the operated leg. In the first measurement, there were value differences of femoral rotation angle between the operated and non-operated limbs from 12° to over 14° for particular phases of the gait cycle. We found excessive internal femoral rotation of the operated limbs and excessive external femoral rotation of the non-operated limbs. At the end of the 14th week after ACLR, in five out of six analysed gait phases the rotation movement patterns in the hip were restored. A significant limitation of movement patterns of external femoral rotation was still maintained at the involved side during the maximal extension of the operated leg in the K4 phase.

The research of the present study showed small and mostly statistically insignificant ETR disorders during gait in the ankle joint of the operated legs. During the 4th week after ACLR in two first phases of gait, a limitation of internal rotation patterns of pelvis in the operated legs was noted. In phases K3 and K4 excessive external rotation patterns of pelvis were noted in the operated legs. During the second and third measurements the values of pelvic kinematic rotation at the operated side were similar to those obtained for the un-operated side.

However, it is difficult to overlook the deficits, such as asymmetry of rotational values for certain phases of gait, in three studied joints of the operated leg compared with the un-operated leg or with the results of the control group, despite the lack of significant differences.

In the summary, it should be noted that the applied physiotherapeutic procedure allowed the patients to regain most of the studied kinematic rotation patterns during the gait on a flat surface in the large joints of the lower limbs and the pelvis between the 4th and the 14th week post-operatively.

Assessing of tibial rotation during gait and all other types of locomotion is usually analysed in short time after ACLR. The early studies on rotation kinematics are justified, given the surgical procedures applied thus far in addition to the current postoperative protocols of physiotherapy. Implementation of closely defined postoperative procedures after ACLR may affect the remote results of gait kinematics [22, 23, 24]. But one of the numerous causes of osteoarthritis is biomechanical overload, affecting the surface of the articular cartilage for many years, especially during various kinds of locomotion [25–27]. We need to require both early and remote studies and analyses. Therefore, acquired knowledge of future development of knee surgery techniques and rehabilitation protocols is important [28–32]. Therefore, the goal of double-bundle reconstruction of the anterior cruciate ligament was to obtain a rotational stability of the tibia better than that achieved using a single-bundle reconstruction. Comparison of double-bundle reconstruction to single-bundle reconstruction results of tibial rotation during gait, as well as kinematic and kinetic parameters of tibial rotation during the dynamic pivot test are not different between methods [20, 23, 33]. Nevertheless, in both groups, the values of tibial rotation were smaller compared with those obtained from un-operated knees and the control group [23]. The influence of the dominant leg only on tibial rotation during gait after ACLR has been analysed [34]. Tibial translation towards the femur, and X-ray images of joints after ACLR have been assessed [35, 36].

The analysis of rotational movement patterns in joints of lower limbs is very important for clinical assessment because of two main reasons. Firstly, because of the most common mechanism of ACL injury (knee excessive rotation, valgus and flexion) and prevention of reinjuries, and secondly, because the main goal of ACL reconstruction is to restore anterior and rotational stability of the tibia against the femur in the knee joint. What’s more, one of the main goals of the postoperative physiotherapeutic procedure is to restore the normal patterns of human locomotion, so gait analysis has to be performed on different stages of physiotherapeutic procedures. The analysis is supposed to provide information not only about the sagittal plane but also about the transverse plane. It also has to involve all of the lower limb joints and pelvis, as it has to be a comprehensive evaluation of all components of normal human gait.

Analysis of rotations occurring only in knee joints does not reflect all of the multiarticular disorders of gait kinematics. The study also suggests that analyzing tibial rotation in the knee joint with simultaneous changes in rotation in large joints of the lower limbs provides better opportunities than singular analysis of rotation in the knee joint for the assessment of disorders in gait kinematics.

In the future, recording kinematic rotation in joints of the lower limbs and pelvis is required. This is necessary for quantitative and qualitative evaluation of biomechanical parameters in patients after ACLR while going up and downstairs, running, and jumping during subsequent stages of physiotherapy [37–41]. This type of protocol could be used for the follow-up assessment of gait parameters and other types of patients locomotion [42]. The effectiveness of applied gait assessment methods during the physiotherapeutic procedure has been confirmed in patients who suffered injuries of other structures of the motor organ. Future studies should assess the risk of recurrent ACL injuries [43].

This study has a limitation, which is the relatively short time of observation. A long term observational study assessing the relationship between changed gait kinematics and the risk of early knee osteoarthritis should be performed. Rotational movement assessment without sagittal plane kinematics has its limitations. This study does not include any results of movement patterns in the sagittal plane due to the fact that they are already well described [1–5, 8]. Another limitation of presented type of assessment is reducing it to the biomechanical assessment only. It should be performed in connection with a comprehensive clinical evaluation, as well as standard assessment scales and functional tests used in treatment of patients after ACLR.

## Conclusions

During gait on a flat surface, evaluation carried out in the 4th, 9th and 14th week of physiotherapy after ACLR, shows a significant improvement of most of the studied kinematic rotation patterns. Between the 9th and 14th weeks following ACLR, there are normal values of foot, tibia, and pelvic rotation in the operated legs compared with results obtained from un-operated legs and the control group. Most of the disorders are found in hip joint rotation at the involved side. These disorders are maintained for a long period of time, and after 14 weeks postoperatively, they are not fully eliminated during maximal extension of the knee during preparation for the stance phase. Analysis of tibial rotation with simultaneous changes in rotation of the large joints in the lower limbs and pelvis during gait provides new cognitive and application data for physicians. These data could enable planning, correction, and assessment of postoperative procedures in ACLR patient.

## References

[1] DeVita P, Hortobagyi T, Barrier J, Torry M, Glover KL, Speroni DL, Money J, Mahar MT (1997). Gait adaptations before and after anterior cruciate ligament reconstruction surgery. Med Sci Sports Exerc.

[2] Knoll Z, Kiss R, Kocsis L (2004). Gait adaptation in ACL deficient patients before and after anterior cruciate ligament reconstruction surgery. J Electromyogr Kinesiol.

[3] DeVita P, Hortobagyi T, Barrier J (1998). Gait biomechanics are not normal after anterior cruciate ligament reconstruction and accelerated rehabilitation. Med Sci Sports Exerc.

[4] Ferber R, Osternig L, Woollacott M, Wasielewski NJ, Lee JH (2002). Gait mechanics in chronic ACL deficiency and subsequent repair. Clin Biomech (Bristol, Avon).

[5] Ferber R, Osternig L, Woollacott M, Wasielewski NJ, Lee JH (2003). Gait perturbation response in chronic anterior cruciate ligament deficiency and repair. Clin Biomech (Bristol, Avon).

[6] Winiarski S, Rutkowska–Kucharska A (2009). Estimated ground reaction force in normal and pathological gait. Acta Bioeng Biomech.

[7] Shorter KA, Polk J, Rosengren KS, Hsiao-Wecksler ET (2008). A new approach to detecting asymmetries in gait. Clin Biomech (Bristol, Avon).

[8] Winiarski S, Czamara A (2012). Evaluation of gait kinematics and symmetry during the first two stages of physiotherapy after anterior cruciate ligament reconstruction. Acta Bioeng Biomech.

[9] Czamara A (2010). Evaluation of physiotherapeutic procedures after ACL reconstruction in males. Archiv Budo.

[10] Georgoulis A, Papadonikolakis A, Papageorgiou C, Mitsou A, Stergiou N (2003). Three-dimensional tibiofemoral kinematics of the anterior cruciate ligament-deficient and reconstructed knee during walking. Am J Sports Med.

[11] Gao B, Zheng N (2010). Alterations in three-dimensional joint kinematics of anterior cruciate ligament-deficient and -reconstructed knees during walking. Clin Biomech (Bristol, Avon).

[12] Miller M, Hart J, MacKnight J (2010). Essential orthopaedics.

[13] Czamara A, Tomaszewski W, Bober T, Lubarski B (2011). The effect of physiotherapy on knee joint extensor and flexor muscle strength after anterior cruciate ligament reconstruction. Med Sci Monit.

[14] BTS Smart Capture User’s Guide. Milan: BTS Bioengeneering; 2006.

[15] Davis R, Ounpuu S, Tyburski D, Gage J (1991). A gait analysis data collection and reduction technique. Hum Mov Sci.

[16] Shrout PE, Fleiss JL (1979). Intraclass correlations: uses in assessing rater reliability. Psychol Bull.

[17] Cicchetti DV, Sparrow SA (1981). Developing criteria for establishing interrater reliability of specific items: applications to assessment of adaptive behavior. Am J Ment Defic.

[18] Razali N, Wah Y (2011). Power comparisons of Shapiro-Wilk, Kolmogorov-Smirnov, Lilliefors and Anderson, darling tests. J Stat Mod Anal.

[19] Royston P, Remark A (1995). R94: remark on algorithm AS181: the W-test for normality. J Roy Stat Soc.

[20] Claes S, Neven E, Callewaert B, Desloovere K, Bellemans J (2011). Tibial rotation in single- and double-bundle ACL reconstruction: a kinematic 3-D *in vivo* analysis. Knee Surg Sports Traumatol Arthrosc.

[21] Czamara A, Markowska I, Królikowska A, Szopa A, Domagalska-Szopa M. Kinematics of rotation in joints of the lower limbs and pelvis during gait: early results. SB ACLR approach versus DB ACLR approach. BioMed Res Int. 2015. doi: 10.1155/2015/707168. 10.1155/2015/707168 PMC4397484 25922839

[22] Sanford BA, Zucker-Levin AR, Williams JL, Mihalko WM, Jacobs EL (2012). Principal component analysis of knee kinematics and kinetics after anterior cruciate ligament reconstruction. Gait Posture.

[23] Webster K, Feller J (2011). Alterations in joint kinematics during walking following hamstring and patellar tendon anterior cruciate ligament reconstruction surgery. Clin Biomech (Bristol, Avon).

[24] Webster K, Feller J, Wittwer J (2012). Longitudinal changes in knee joint biomechanics during level walking following anterior cruciate ligament reconstruction surgery. Gait Posture.

[25] Andriacchi T, Koo S, Scanlan S (2009). Gait mechanics influence healthy cartilage morphology and osteoarthritis of the knee. J Bone Joint Surg Am.

[26] Astephen JL, Deluzio KJ, Caldwell GE, Dunbar MJ (2008). Biomechanical changes at the hip, knee, and ankle joints during gait are associated with knee osteoarthritis severity. J Orthop Res.

[27] Bejek Z, Paróczai R, Illyés A, Kiss R (2006). The influence of walking speed on gait parameters in healthy people and in patients with osteoarthritis. Knee Surg Sports Traumatol Arthrosc.

[28] Hertel P, Behrend H, Cierpinski T, Musahl V, Widjaja G (2005). ACL reconstruction using bone-patellar tendon-bone press-fit fixation: 10-year clinical results. Knee Surg Sports Traumatol Arthrosc.

[29] Misonoo G, Kanamori A, Ida H, Miyakawa S, Ochiai N (2012). Evaluation of tibial rotational stability of single-bundle vs. anatomical double-bundle anterior cruciate ligament reconstruction during a high-demand activity - a quasi-randomized trial. Knee.

[30] Scanlan SF, Chaudhari AM, Dyrby CO, Andriacchi TP (2010). Differences in tibial rotation during walking in ACL reconstructed and healthy contralateral knees. J Biomech.

[31] Widuchowski W, Widuchowska M, Koczy B, Dragan S, Czamara A, Tomaszewski W, Widuchowski J (2012). Femoral press-fit fixation in ACL reconstruction using bone-patellar tendon- bone autograft: results at 15 years follow-up. BMC Musculoskelet Discord.

[32] Zabala M, Favre J, Scanlan S, Donahue J, Andriacchi TP (2013). Three-dimensional knee moments of ACL reconstructed and control subjects during gait, stair ascent, and stair descent. J Biomech.

[33] Tsarouhas A, Losifidis M, Kotzanitelos D, Spyropoulos G, Tsatalas T, Giakas G (2010). Three- dimensional kinematic and kinetic analysis of knee rotational stability after single- and double –bundle anterior cruciate ligament reconstruction. Arthroscopy.

[34] Wang H, Fleischli J, Nigel Z (2012). Effect of lower limb dominance on knee joint kinematics after anterior cruciate ligament reconstruction. Clin Biomech (Bristol, Avon).

[35] Hart JM, Blanchard BF, Hart JA, Montgomery SC, Schoderbek R, Miller MD (2009). Multiple ligament knee reconstruction clinical follow-up and gait analysis. Knee Surg Sports Traumatol Arthrosc.

[36] Tagesson S, Oberg B, Kvist J. Static and dynamic tibial translation before, 5 weeks after, and 5 years after anterior cruciate ligament reconstruction. Knee Surg Sports Traumatol Arthrosc. 2014. [Epub ahead of print]. 10.1007/s00167-014-3279-8 25261221

[37] Chouliaras V, Ristanis S, Moraiti C, Tzimas V, Stergiou N, Georgoulis AD (2009). Anterior cruciate ligament reconstruction with a quadrupled hamstrings tendon autograft does not restore tibial rotation to normative levels during landing from a jump and subsequent pivoting. J Sports Med Phys Fitness.

[38] Chouliaras V, Ristanis S, Moraiti C, Stergiou N, Georgoulis AD (2007). Effectiveness of reconstruction of the anterior cruciate ligament with quadrupled hamstrings and bone-patellar tendon-bone autografts: an *in vivo* study comparing tibial internal-external rotation. Am J Sports Med.

[39] Georgoulis A, Ristanis S, Chouliaras V, Moraiti C, Stergiou N (2007). Tibial rotation is not restored after ACL reconstruction with a hamstring graft. Clin Orthop Relat Res.

[40] Lewek M, Rudolph K, Axe M, Snyder-Mackler L (2002). The effect of insufficient quadriceps strength on gait after anterior cruciate ligament reconstruction. Clin Biomech (Bristol, Avon).

[41] Tashman S, Collon D, Anderson K, Kolowich P, Anderst W (2004). Abnormal rotational knee motion during running after anterior cruciate ligament reconstruction. Am J Sports Med.

[42] White K, Di Stasi SL, Smith AH, Snyder-Mackler L (2013). Anterior cruciate ligament – specialized post-operative return-to-sports (ACL-SPORTS) training: a randomized control trial. BMC Musculoskelet Disord.

[43] Delahunt E, Prendiville A, Sweeney L, Chawke M, Kelleher J, Patterson M, Murphy K (2012). Hip and knee joint kinematics during a diagonal jump landing in anterior cruciate ligament reconstructed females. J Electromyogr Kinesiol.

